# Supplementary material for: MetaRibo-Seq measures translation in microbiomes
Source: Nat Commun. 2020 Jun 29;11:3268. doi: 10.1038/s41467-020-17081-z (PMC7324362; doi:10.1038/s41467-020-17081-z)
Supplement: Supplementary file 10 — Supplementary Data 7 [file 41467_2020_17081_MOESM10_ESM.zip › File2/Confidence_High_Taxonomy/223102_out.krona.html]

Javascript must be enabled to view this page.

members
magnitude
magnitudeUnassigned
count
unassigned
taxon
rank

223102\_out

3

superkingdom
2
3

3
1239
phylum

3
186801
class

3
186802

SRS014235\_contig\_number\_contig-100\_16229.149853
order
1

family
541000
2

species
2
1898205

SRS019685\_contig\_number\_24344SRS049446\_contig\_number\_4259
